# BNP and Admission Glucose as In-Hospital Mortality Predictors in Non-ST Elevation Myocardial Infarction

**DOI:** 10.1100/2012/397915

**Published:** 2012-02-01

**Authors:** Julio Yoshio Takada, Rogério Bicudo Ramos, Solange Desiree Avakian, Soane Mota dos Santos, José Antonio Franchini Ramires, Antonio de Pádua Mansur

**Affiliations:** ^1^Heart Institute (InCor), University of São Paulo Medical School, Avenue Enéas de Carvalho de Aguiar 44, 05403-000 São Paulo, SP, Brazil; ^2^Institute of Mathematics and Statistics, University of São Paulo, R. do Matão 1010, 05508-090 São Paulo, SP, Brazil

## Abstract

*Objectives*. Admission hyperglycemia and B-type natriuretic peptide (BNP) are associated with mortality in acute coronary syndromes, but no study compares their prediction in-hospital death. *Methods*. Patients with non-ST-elevation myocardial infarction (NSTEMI), in-hospital mortality and two-year mortality or readmission were compared for area under the curve (AUC), sensitivity (SEN), specificity (SPE), positive predictive value (PPV), negative predictive value (NPV), and accuracy (ACC) of glycemia and BNP. *Results*. Respectively, AUC, SEN, SPE, PPV, NPV, and ACC for prediction of in-hospital mortality were 0.815, 71.4%, 84.3%, 26.3%, 97.4%, and 83.3% for glycemia = 200 mg/dL and 0.748, 71.4%, 68.5%, 15.2%, 96.8% and 68.7% for BNP = 300 pg/mL. AUC of glycemia was similar to BNP (*P* = 0.411). In multivariate analysis we found glycemia ≥200mg/dL related to in-hospital death (*P* = 0.004). No difference was found in two-year mortality or readmission in BNP or hyperglycemic subgroups. *Conclusion*. Hyperglycemia was an independent risk factor for in-hospital mortality in NSTEMI and had a good ROC curve level. Hyperglycemia and BNP, although poor in-hospital predictors of unfavorable events, were independent risk factors for death or length of stay >10 days. No relation was found between hyperglycemia or BNP and long-term events.

## 1. Introduction

In recent years, incorporation of new laboratory methods in clinical practice has improved diagnosis and prognostic prediction of outcomes in acute coronary syndromes (ACS). Cardiac troponins, with high sensitivity and specificity for diagnosing myocardial injury [1], and B type natriuretic factor (BNP), primarily used in acute decompensation of heart failure but with prognostic significance in ACS [2], are useful in the initial evaluation of patients with acute chest pain [3–5]. Glycemia, determined by using a simple, low-cost laboratory test, has been associated with a worse prognosis in ACS patients, even in the absence of diabetes [6].

Studies have shown BNP as a prognostic factor for both early and late outcomes in non-ST elevation ACS, a presumed reflection of left ventricular dysfunction [7]. Non-ST elevation myocardial infarction (NSTEMI) has lower in-hospital mortality than ST elevation myocardial infarction has, but higher unadjusted events in years following hospitalization [8]. So, we hypothesized that hyperglycemia and BNP could predict patients at risk for both in-hospital and 2-year events.

## 2. Methods

### 2.1. Study Design and Population

Patients with ACS admitted consecutively between January 2005 and April 2006 to an urban academic cardiology emergency single center in São Paulo, Brazil were prospectively evaluated with their data registered in the institution's database. Baseline clinical and admission laboratory characteristics, CAD risk factors, medicines used, in-hospital outcomes, angiography, and treatments were observed. The Ethics Committee of the hospital approved this study. A total of 1304 ACS patients were included, and a subgroup of 170 NSTEMI-only patients with admission BNP were followed up for two years. Inclusion criteria were ACS hospital admission, according to the international consensus definition [9], age older than 18 years, and willing to provide written informed consent. Exclusion criteria were non-NSTEMI ACS and incomplete laboratory data. Completed data were available for 96 NSTEMI patients, and written informed consent was obtained from all participants.

### 2.2. Definitions and Data Collection

Clinical outcomes were defined as death, or death and prolonged length of stay (>10 days; mean of length of stay). Length of stay was defined as an outcome, because 90% of patients at our emergency department undergo angiographic study in 48 hours. In this setting, reinfarction or refractory angina are infrequent events at hospitalization. Mean length of stay was 10.5 and median 3.8 days (range: 0.4 to 124.6), and follow-up mean was 21.7 and median 23.2 months (range: 0.11 to 31.7), until September 2007. Long-term outcomes included death or death and hospital readmission for all causes.

Blood samples were collected at admission to the emergency department. For BNP determination, a specific kit for the ADVIA Centaur analyzer (Bayer Health Care Diagnostics, Tarrytown, NY) was used, with detection limit of 2 pg/mL and range from 2 to 5000 pg/mL. The intra- and inter-assay coefficients of variation ranged from 2.1 to 4.7% for concentrations between 29 and 1700 pg/mL. Echocardiographic data were collected in second day of admission and ejection fraction was obtained. We calculated the best point of BNP in receiver operating characteristic (ROC) curve equal to 300 pg/mL, the highest point of Youden's Index [10]. We selected the level of hyperglycemia above 200 mg/dL to separate the highest stratum seen in the literature [11] for admission nonfasting blood glucose. Previous use of medication were assessed.

### 2.3. Statistical Analysis

All data are described as rates and frequencies or means with standard deviations, as appropriate. Differences in the distribution of selected characteristics between patient groups were examined using the chi-square test and Fisher's exact test for categorical variables. The analysis was performed using the Student's *t*-test for normally distributed continuous variables and the Mann-Whitney and Kruskal-Wallis tests for nonparametric variables. Pearson correlation coefficients were used to study the correlations. We built an adjusted model in multivariate regression to analyze the independent variables associated with in-hospital death or combined in-hospital death and length of stay > 10 days: age, hypertension, smoking, diabetes, blood glucose < or ≥ 200 mg/dL, BNP < or ≥ 300 pg/mL, sex, and serum creatinine. The ROC curve was used to determine the area under the curve (AUC) of the C statistic. Cox regression model was used to evaluate independent prognostic factors of mortality and hospital readmission for all causes in the follow-up period. Two-sided *P* values < 0.05 were considered statistically significant. All statistical analyses were performed using SAS software version 9.2 for Windows. To obtain Youden's Index, AUC, sensitivity (SENS), specificity (SPEC), positive predictive value (PPV), negative predictive value (NPV), and accuracy (ACCU), we used a macro of the SAS software [12].

## 3. Results

### 3.1. Admission Blood Glucose Endpoints

Table 1 presents clinical and demographic patient characteristics, grouped by blood glucose < or ≥ 200 mg/dL. Univariate analysis showed a predominance of women (73.7% versus 23.4%; *P* < 0.001), lower levels of chest pain (68.4% versus 92.2%; *P* = 0.005), serum hemoglobin (11.8 versus 13.9 g/dL; *P* < 0.001), coronary angiographic study (73.7% versus 93.5%; *P* = 0.011), and higher levels of diabetes (94.7% versus 38.9%; *P* < 0.001), heart rate (102.7 versus 77.3 beats/minute; *P* < 0.001), BNP (660.8 versus 283.7 pg/mL; *P* < 0.001), white blood cell count (11,722.2 versus 9,437.3 cells/mm^3^; *P* = 0.010), platelet count (294,889.0 versus 221,118.0 cells/mm^3^; *P* < 0.001), serum urea (65.5 versus 49.5 mg/dL; *P* = 0.033), serum creatinine (1.81 versus 1.20 mg/dL; *P* = 0.016), Killip presentation ≥ 2 (47.4% versus 13.0%; *P* < 0.001), length of stay (22.8 versus 7.4 days; *P* < 0.001), in-hospital mortality (26.3% versus 2.6%; *P* < 0.001), and combined prolonged length of stay or death (63.2% versus 16.9%; *P* < 0.001) in the ≥200 mg/dL blood glucose group.

### 3.2. BNP Endpoints

Table 2 presents clinical and demographic patient characteristics, but grouped by BNP < or ≥ 300 pg/mL. The BNP ≥ 300 pg/mL group were older (69.2 versus 62.9 years; *P* = 0.012) and had lower levels of chest pain (66.7% versus 98.4%; *P* < 0.001), systolic blood pressure (125.6 versus 144.5 mmHg; *P* = 0.005), diastolic blood pressure (75.2 versus 87.7 mmHg; *P* < 0.001), hemoglobin (12.4 versus 14.1 g/dL; *P* < 0.001), ejection fraction (37.9% versus 49.3%; *P* = 0.001), coronary angiography study (75.8% versus 96.8%; *P* = 0.001), and higher levels of diabetes (69.7% versus 39.7%; *P* = 0.005), heart rate (94.9 versus 75.6 beats/minute; *P* = 0.001), serum urea (67.5 versus 44.8 mg/dL; *P* < 0.001), serum creatinine (1.76 versus 1.11 mg/dL; *P* = 0.002), blood glucose (213.9 versus 120.2 mg/dL; *P* < 0.001), Killip presentation ≥ 2 (45.5% versus 6.3%; *P* < 0.001), medical therapy alone (51.5% versus 28.6%; *P* = 0.027), length of stay (16.4 versus 7.4 days; *P* = 0.022), in-hospital mortality (15.2% versus 3.2%; *P* = 0.032), and combined prolonged length of stay or death (48.5% versus 14.3%; *P* < 0.001). No difference was found in two-year mortality or readmission for all causes in BNP or hyperglycemic subgroups.

### 3.3. Combination of Laboratory Factors

**Figure 1:**
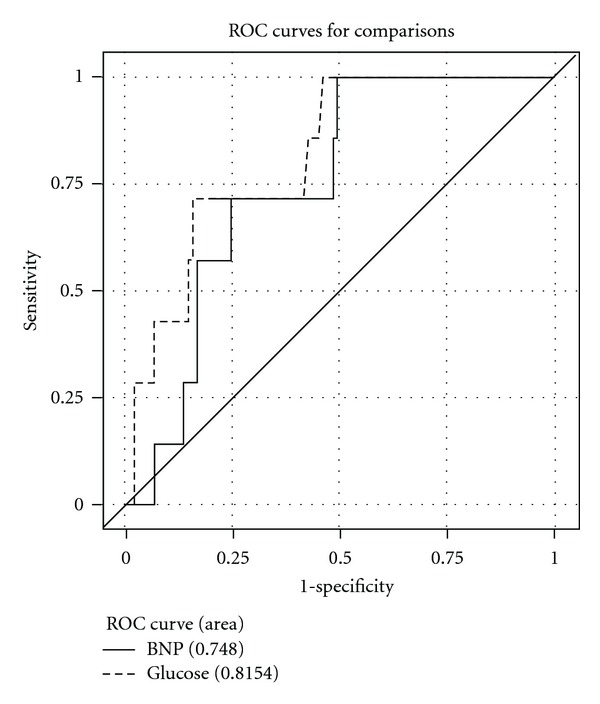
Comparison of glucose versus BNP ROC curve.

**Figure 2:**
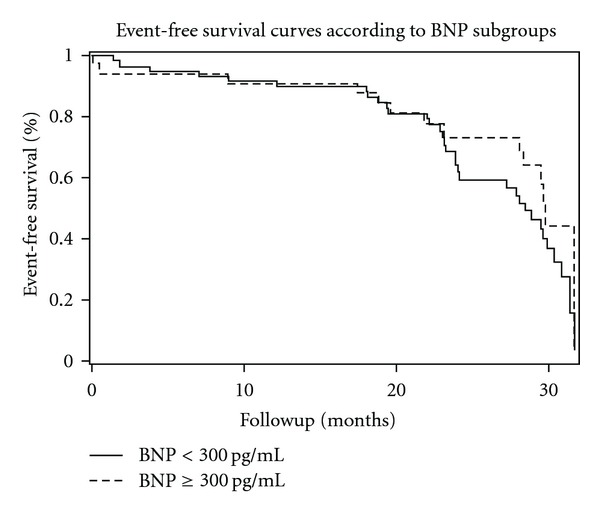
Adjusted two-year event-free survival, according to BNP < or ≥ 300 pg/mL.

In multivariate logistic analysis of the in-hospital period, the adjusted model showed only hyperglycemia ≥ 200 mg/dL as an independent predictor of mortality (OR = 13.036, IC 95% 2.296–74.022; *P* < 0.001). Hyperglycemia ≥ 200 mg/dL (OR = 4.588; IC 95% 1.348–15.610; *P* < 0.001) and BNP ≥ 300 pg/mL (OR = 3.366, IC 95% 1.108–10.223; *P* = 0.027) were independent predictors of length of stay longer than 10 days or death.  Table 3 summarizes AUC, sensitivity, specificity, positive predictive value, negative predictive value, and accuracy of blood glucose and BNP for in-hospital death prediction, and Figure 1 shows ROC curve comparison of only blood glucose and BNP. Although blood glucose had been slightly better, there was no difference between admission glucose and BNP in in-hospital NSTEMI death prediction. In long-term followup, Cox regression (Figure 2) failed to show any independent predictors of mortality or combined hospital readmission for all causes or death. We analyzed the correlation between blood glucose and BNP, as shown in Table 4. There is a significant correlation between glycemia and BNP (Pearson correlation coefficient (PCC) = 0.38; *P* < 0.001), glycemia and hemoglobin (PCC = 0.41; *P* < 0.001), BNP and creatinine (PCC = 0.29; *P* = 0.004), and BNP and hemoglobin (PCC = 0.34; *P* < 0.001).

## 4. Discussion

### 4.1. Hyperglycemia and BNP Mortality of ACS

Hyperglycemia is a factor long associated with ACS mortality [13], and its capacity for predicting a worse prognosis in NSTEMI, in our study, is at least comparable to that of BNP or better if we consider only death as the outcome. Several studies accessing BNP and admission glucose separately in ACS patients showed in-hospital and long-term prognosis around the world [14–19]. The decision to use blood glucose or BNP laboratory results instead of multifactorial models can be more useful for emergency physicians.

Admission hyperglycemia could be a biochemical marker for unfavorable ACS prognosis only reflecting inappropriate diabetes control, as another risk factor, and reflecting advanced atherosclerosis. In fact, hyperglycemic ACS patients usually have a history of diabetes and elevated HbA1C [20]. On the other hand, hyperglycemia could be a signal of adrenergic stress response [21] of a severely ill ACS patient, as observed in other acute illnesses [22]. However, some studies with cardiac magnetic resonance [23, 24] have shown that hyperglycemic or diabetic patients have greater myocardial infarct size and greater microvascular obstruction, prothrombotic state, or endothelial dysfunction [25], or in other words, hyperglycemia could be itself involved in mechanisms of impaired blood nutrition to the ischemic wall.

### 4.2. Marker or Risk Factor?

Hyperglycemia could itself damage coronary endothelia, but there are no convincing data supporting strategies of intensive insulin therapy to control hyperglycemia in critically ill or myocardial infarcted patients that could reverse the increased mortality, most probably due to hypoglycemic risk [26], another great mortality risk factor in the hospital setting. In this context, both blood glucose and BNP are better as prognostic markers in ACS patients. For example, elevated BNP can predict anterior wall ischemic localization at angiography in NSTEMI [27]. 

In our study, BNP and blood glucose were better markers of in-hospital outcomes. Admission hyperglycemia is associated with long-term risk for ACS mortality [28], but this association is not homogeneous in different ACS presentations [15], unstable angina, NSTEMI, or STEMI. Besides, mortality up to 1 year can be predicted both by admission glucose and fasting blood glucose, but the better predictor of mortality for longer periods is fasting glucose [29]. 

Correlations between BNP, glycemia, hemoglobin, and creatinine illustrated how measurement of one laboratory element can in fact assess other components of body homeostasis, becoming more difficult to separate a few independent mortality risk factors in adjusted complex models. These factors may be not related in a particular patient, or all factors could exist in consequence of a common combination of diseases: obesity, hypertension and diabetes leading to kidney disease, coronary disease, heart failure, anemia, elevated BNP.

In-hospital stay was defined by clinical criteria. More severe patient were related to in-hospital mortality or required more time to be discharged from hospital. Hyperglycemia and BNP are markers of this severity of illness. 

### 4.3. ROC Curve

At a cutoff point of 200 mg/dL for blood glucose and 300 pg/dL for BNP, ROC curve analysis showed modest values of sensitivity and specificity, but a great negative predictive value for mortality. Eggers et al. [30] compared NT-pro BNP, CRP, cystatin C, and creatinine clearance to predict reinfarction or death in patients with chest pain and ACS non-STEMI. They found AUC = 0.80 in a composite of abnormal EKG, increased cardiac troponin I and NT-pro BNP, best NT-pro BNP cutoff  point = 550 ng/L, sensitivity around 77%, and specificity around 20%, according to the ROC curve. Correia et al. [31] studied NSTEMI patients and found no improvement in the Grace Score prediction model of in-hospital events with addition of admission glucose to the model: AUC increased from 0.81 to 0.82. 

The comparison between admission blood glucose and BNP ROC curves showed no statistical difference, but we believe that blood glucose could be more useful in risk stratification of ACS patients mainly in undeveloped countries, where cost limits the adoption of new technologies. 

### 4.4. Study Limitations

We determined admission laboratory results as variables to be studied to evaluate risks beyond clinical scores like Grace or TIMI risk. We did not compare or add BNP and blood glucose to the models of risk prediction, but we simplified NSTEMI assessment by using unadjusted results to predict mortality or higher length of stay. 

Some aspects of the study population like prevalence of previous coronary artery disease in up to three-quarters of patients could influence initial BNP or blood glucose and the in-hospital prognosis. 

## 5. Conclusion

This study suggests that hyperglycemia is an independent risk factor of in-hospital mortality in NSTEMI and has a good ROC curve level. Although poor in-hospital predictors of unfavorable events, both hyperglycemia and BNP were independent risk factors for death or length of stay > 10 days. There was no relation between hyperglycemia or BNP with long-term events in our NSTEMI patients. 

## Figures and Tables

**Table 1:** Clinical and demographic patient characteristics, grouped by blood glucose ≥ or < 200 mg/dL.

	All	Glucose < 200 mg/dL	Glucose ≥ 200 mg/dL	*P*
Patients, *n* (%)	96	77 (80.2)	19 (19.8)	
Age (years)*	65.1 (11.7)	64.9 (12.0)	65.7 (10.7)	0.791
Male, *n* (%)	64 (66.7)	59 (76.6)	5 (26.3)	<0.001
Prior coronary artery disease, *n* (%)	57 (59.4)	43 (55.8)	14 (73.7)	0.156
Hypertension, *n* (%)	86 (89.6)	67 (87.0)	19 (100)	0.097
Current smoker, *n* (%)	16 (16.7)	13 (16.7)	3 (15.8)	0.909
Dyslipidemia, *n* (%)	65 (67.7)	53 (68.8)	12 (63.2)	0.636
Diabetes mellitus, *n* (%)	48 (50)	30 (38.9)	18 (94.7)	<0.001
Family history of coronary artery disease, *n* (%)	35 (36.5)	29 (37.7)	6 (31.6)	0.622
Heart rate (beats/minute)	82.6 (27.6)	77.3 (71.9)	102.7 (86.4)	<0.001
Systolic blood pressure (mmHg)	137.5 (30.8)	140.2 (29.6)	127.8 (33.7)	0.121
Diastolic blood pressure (mmHg)	83.1 (17.1)	84.8 (16.6)	76.8 (17.6)	0.072
Killip > I, *n* (%)	19 (19.8)	10 (13.0)	9 (47.4)	<0.001
Admission glucose (mg/dL)	152.4 (100.0)	113.1 (31.8)	311.9 (122.8)	<0.001
BNP (pg/mL)	358.3 (381.0)	283.7 (339.3)	660.8 (399.4)	<0.001
Hemoglobin (g/dL)	13.5 (2.1)	13.9 (1.9)	11.8 (2.2)	<0.001
White blood cell count (/mm3)	9879.6 (3418.4)	9437.3 (3181.0)	11722.2 (3838.3)	0.010
Platelet count (/mm3)	235244.7 (82837.8)	221118.0 (73987.4)	294889.0 (93436.3)	<0.001
Serum urea (mg/dL)	52.7 (29.4)	49.5 (25.9)	65.5 (38.6)	0.033
Serum creatinine (mg/dL)	1.33 (1.00)	1.20 (0.48)	1.81 (1.98)	0.016
Creatinine kinase-MB (CKMB) peak (ng/mL)	65.1 (111.1)	73.2 (119.5)	32.5 (58.7)	0.154
Troponin I (ng/mL)	25.8 (40.6)	26.5 (41.9)	23.1 (35.6)	0.741
Cholesterol (mg/dL)	175.0 (39.4)	174.7 (39.7)	176.4 (39.3)	0.892
HDL-C (mg/dL)	41.0 (10.9)	40.9 (10.9)	41.5 (11.2)	0.872
LDL-C (mg/dL)	110.6 (32.5)	110.0 (32.9)	113.4 (321.2)	0.732
Triglycerides (mg/dL)	130.4 (76.2)	129.2 (72.4)	136.0 (95.9)	0.772
Ejection fraction (%)	44.7 (15.3)	46.4 (14.3)	39.4 (17.4)	0.083
Angiography, *n* (%)	86 (89.6)	72 (93.5)	14 (73.7)	0.011
Medical therapy, *n* (%)	35 (36.5)	26 (33.8)	9 (47.4)	0.270
Percutaneous coronary intervention, *n* (%)	49 (57.0)	43 (59.7)	6 (42.9)	0.243
Surgery, *n* (%)	12 (12.5)	8 (10.4)	4 (21.0)	0.208
Length of stay (days)	10.5 (18.6)	7.4 (12.9)	22.8 (30.2)	<0.001
Followup (months)	21.7 (9.7)	22.5 (8.7)	18.2 (12.8)	0.084
Mortality, *n* (%)	17 (17.7)	11 (14.3)	6 (31.6)	0.077
In-hospital, *n* (%)	7 (7.3)	2 (2.6)	5 (26.3)	<0.001
Followup, *n* (%)	10 (11.2)	9 (12.0)	1 (7.1)	0.597
In-hospital mortality + prolonged length of stay, *n* (%)	25 (26.0)	13 (16.9)	12 (63.2)	<0.001
Follow-up mortality + readmission, *n* (%)	51 (57.3)	43 (57.3)	8 (57.1)	0.989

**Table 2:** Clinical and demographic patient characteristics, grouped by BNP ≥ or < 300 pg/mL.

	All	BNP < 300 pg/mL	BNP ≥ 300 pg/mL	*P*
Patients, *n* (%)	96	63 (65.6)	33 (34.4)	
Age (years)*	65.1 (11.7)	62.9 (11.1)	69.2 (12.0)	0.012
Male, *n* (%)	64 (66.7)	46 (73.0)	18 (54.5)	0.068
Prior coronary artery disease, *n* (%)	57 (59.4)	34 (54.0)	23 (69.7)	0.136
Hypertension, *n* (%)	86 (89.6)	54 (85.7)	32 (97.0)	0.086
Current smoker, *n* (%)	16 (16.7)	12 (19.0)	4 (12.1)	0.387
Dyslipidemia, *n* (%)	65 (67.7)	43 (68.3)	22 (66.7)	0.874
Diabetes mellitus, *n* (%)	48 (50)	25 (39.7)	23 (69.7)	0.005
Family history of coronary artery disease, *n* (%)	35 (36.5)	25 (39.7)	10 (30.3)	0.365
Heart rate (beats/minute)	82.6 (27.6)	75.6 (21.8)	94.9 (32.4)	0.001
Systolic blood pressure (mmHg)	137.5 (30.8)	144.5 (29.7)	125.6 (29.3)	0.005
Diastolic blood pressure (mmHg)	83.1 (17.1)	87.7 (16.7)	75.2 (14.9)	<0.001
Killip > I, *n* (%)	19 (19.8)	4 (6.3)	15 (45.5)	<0.001
Admission glucose (mg/dL)	152.4 (100.0)	120.2 (46.9)	213.9 (139.6)	<0.001
Admission glucose ≥ 200 mg/dL, *n* (%)	19 (19.8)	4 (6.3)	15 (45.5)	<0.001
BNP (pg/mL)	358.3 (381.0)	142.9 (87.6)	769.6 (387.5)	<0.001
Hemoglobin (g/dL)	13.5 (2.1)	14.1 (1.8)	12.4 (2.2)	<0.001
White blood cell count (/mm3)	9879.6 (3418.4)	9459.0 (3166.8)	10681.3 (3775.9)	0.102
Platelet count (/mm3)	235244.7 (82837.8)	232967.0 (71510.4)	239455.0 (101620.0)	0.719
Serum urea (mg/dL)	52.7 (29.4)	44.8 (19.0)	67.5 (38.7)	<0.001
Serum creatinine (mg/dL)	1.33 (1.00)	1.11 (0.39)	1.76 (1.55)	0.002
Creatinine kinase-MB (CKMB) peak (ng/mL)	65.1 (111.1)	60.1 (99.9)	74.7 (131.0)	0.542
Troponin I (ng/mL)	25.8 (40.6)	20.0 (32.7)	36.9 (51.2)	0.056
Cholesterol (mg/dL)	175.0 (39.4)	180.0 (37.9)	165.5 (41.1)	0.122
HDL-C (mg/dL)	41.0 (10.9)	40.9 (10.3)	41.3 (12.1)	0.886
LDL-C (mg/dL)	110.6 (32.5)	114.0 (31.3)	103.7 (34.2)	0.190
Triglycerides (mg/dL)	130.4 (76.2)	129.9 (65.3)	131.1 (94.5)	0.947
Ejection fraction (%)	44.7 (15.3)	49.3 (14.6)	37.9 (13.9)	0.001
Angiography, *n* (%)	86 (89.6)	61 (96.8)	25 (75.8)	0.001
Medical therapy, *n* (%)	35 (36.5)	18 (28.6)	17 (51.5)	0.027
Percutaneous coronary intervention, *n* (%)	49 (57.0)	38 (62.3)	11 (44.0)	0.119
Surgery, *n* (%)	12 (12.5)	7 (11.1)	5 (15.2)	0.570
Length of stay (days)	10.5 (18.6)	7.4 (14.7)	16.4 (23.5)	0.022
Followup (months)	21.7 (9.7)	22.7 (8.3)	19.7 (11.9)	0.143
Mortality, *n* (%)	17 (17.7)	8 (12.7)	9 (27.3)	0.076
In-hospital, *n* (%)	7 (7.3)	2 (3.2)	5 (15.2)	0.032
Followup, *n* (%)	10 (11.2)	6 (9.8)	4 (14.3)	0.537
In-hospital mortality + prolonged length of stay, *n* (%)	25 (26.0)	9 (14.3)	16 (48.5)	<0.001
Follow-up mortality + readmission, *n* (%)	51 (57.3)	36 (59.0)	15 (53.4)	0.630

**Table 3:** Comparison of BNP and blood glucose in outcome predictions. Area under the curve, sensitivity, specificity, positive predictive value, negative predictive value, and accuracy.

Biochemical factor	Point	AUC*	Sensitivity (%)	Specificity (%)	PPV (%)*	NPV (%)*	Accuracy (%)	*P* ^†^
In-hospital mortality								0.411
Glucose (mg/dL)	200	0.819	71.4	84.3	26.3	97.4	83.3	
BNP (pg/mL)	300	0.748	71.4	68.5	15.2	96.8	68.7	

Combined length of stay ≥ 10 days or in-hospital mortality								0.339
Glucose (mg/dL)	200	0.697	48.0	90.1	63.2	83.1	79.2	
BNP (pg/mL)	300	0.760	64.0	76.1	48.5	85.7	72.9	

Combined hospital readmission or mortality								0.343
Glucose (mg/dL)	200	0.524	15.7	84.3	42.9	57.3	55.1	
BNP (pg/mL)	300	0.4631	34.2	70.6	46.4	59	55.1	

*AUC: area under the curve; PPV: positive predictive value; NPV: negative predictive value; ^†^comparison AUC admission blood glucose versus BNP.

**Table 4:** Correlations between blood glucose, BNP, hemoglobin, and creatinine.

Factor 1	Factor 2	*P* value	Pearson Correlation Coefficient
Glycemia	BNP	<0.001	0.381
Glycemia	Creatinine	0.140	0.153
Glycemia	Hemoglobin	<0.001	0.413
BNP	Creatinine	0.004	0.292
BNP	Hemoglobin	<0.001	−0.348
Hemoglobin	Creatinine	0.008	−0.273
